# Effects of Dapagliflozin on Obese Patients With Type 2 Diabetes: A Prospective Observational Study From Bangladesh

**DOI:** 10.7759/cureus.89360

**Published:** 2025-08-04

**Authors:** Somya Binte Akhond, Jamila Bupasha, Gull E Jannat, Lubna Sharmin, Md Nazmul Hossain Sumon, Ruma Akhter, Fahim Mahbub

**Affiliations:** 1 Department of Respiratory Medicine, Mukti Hospital, Cumilla, BGD; 2 Department of Surgery, Enam Medical College and Hospital, Dhaka, BGD; 3 Department of Dermatology, Ashiyan Medical College and Hospital, Dhaka, BGD; 4 Department of Medicine, Kabir's Healthcare Hospital, Dhaka, BGD; 5 Department of Surgery, The Royal Private Hospital, Moulvibazar, BGD; 6 Department of General Internal Medicine, Al-Reza General Hospital, Jamalpur, BGD; 7 Department of Medicine, Bangladesh Institute of Health Sciences (BIHS) General Hospital, Dhaka, BGD

**Keywords:** dapagliflozin, diabetes mellitus type 2, glycated hemoglobin, lipid profile, obesity

## Abstract

Objective: Type 2 diabetes mellitus (T2DM) is a chronic metabolic disorder frequently associated with obesity, leading to increased risks of cardiovascular and renal complications. Dapagliflozin, a sodium-glucose cotransporter-2 (SGLT2) inhibitor, has emerged as a promising therapeutic agent for improving glycemic control and promoting weight reduction. However, evaluating its safety and efficacy in obese T2DM patients remains essential, particularly in real-world clinical settings. This study aimed to assess the safety and efficacy of dapagliflozin in obese patients with type 2 diabetes mellitus (T2DM) by assessing adverse event profiles and the reduction in HbA1c levels over the treatment period.

Methodology: This 12-month prospective, multicenter observational study was conducted from April 2023 to April 2024 at BIRDEM (Bangladesh Institute of Research and Rehabilitation in Diabetes, Endocrine and Metabolic Disorders) General Hospital, Dhaka, and affiliated healthcare centers in Bangladesh. One thousand five hundred patients with type 2 diabetes mellitus and a body mass index (BMI) ≥30 kg/m² were consecutively enrolled from outpatient clinics. Eligibility was based on a confirmed diagnosis of type 2 diabetes and the recent initiation of dapagliflozin therapy. Patients received 5 mg or 10 mg of dapagliflozin daily, either as monotherapy or in combination with other antidiabetic agents. Physicians made all treatment decisions independently as part of routine clinical care. Clinical and laboratory data were extracted from medical records using a standardized case record form. Outcomes included changes in glycemic and lipid parameters at baseline, three, six, and 12 months, and the frequency of adverse events. Statistical analyses were performed using IBM SPSS Statistics for Windows, version 25 (IBM Corp., Armonk, NY), with statistical significance set at p < 0.05.

Results: The study showed a statistically significant reduction in mean glycated hemoglobin levels from 8.1 ± 1.7 at baseline to 7.3 ± 1.4 after six months of treatment (p<0.0001). Adverse events were reported in 240 (16%) patients, with the most common being 90 (37.5%) cases of fatigue and hypoglycemia each. Urinary tract infection was observed in 60 (25%) cases, vulvovaginal pruritus and dysuria in 30 (12.5%) cases. A total of 30 (12.5%) patients developed diabetic ketoacidosis (DKA), primarily those with a long-standing history of diabetes (≥10 years) and prior hypertension.

Conclusion: Dapagliflozin appears to be an effective therapeutic option for the management of type 2 diabetes in obese individuals, contributing to improvements in glycemic control and metabolic parameters. When administered alone or in combination with other antidiabetic agents, it was associated with favorable clinical outcomes and an acceptable safety profile, supporting its utility in routine clinical practice.

## Introduction

Type 2 diabetes mellitus (T2DM) is characterized by chronic hyperglycemia resulting from impaired insulin production, insulin resistance in peripheral tissues, or a combination of both. A key feature of T2DM is the impaired metabolism of carbohydrates, lipids, and proteins. Globally, over 463 million individuals had diabetes in 2019, and projections indicate that this number will rise to nearly 640 million by 2040 [1]. Among adults, T2DM is the most prevalent form of diabetes, accounting for approximately 90% of all reported cases [2]. Individuals with diabetes have a markedly increased risk of cardiovascular and renal complications, along with higher mortality rates than the general population [3].

Effective glycemic control in T2DM can often be achieved through non-pharmacological interventions, such as lifestyle modifications or bariatric surgery [4]. Although lifestyle modifications and bariatric surgery can help some patients achieve glycemic control, many individuals with T2DM still require pharmacological treatment to maintain optimal blood glucose levels. Currently, pharmacological therapies for T2DM focus on improving insulin sensitivity, delaying carbohydrate absorption in the gastrointestinal tract, stimulating insulin secretion, or increasing glucose excretion via sodium-glucose cotransporter-2 (SGLT2) inhibitors [5]. Compared to other SGLT2 inhibitors, such as empagliflozin and canagliflozin, dapagliflozin has a broader impact on heart failure and chronic kidney disease outcomes, making it a valuable option beyond glycemic control [3]. By inhibiting sodium reabsorption, dapagliflozin also decreases the ventricular preload and afterload, reducing sympathetic activity by increasing sodium delivery to the kidney’s distal tubules [6].

Dapagliflozin is approved for the treatment of poorly controlled T2DM in adults. It is recommended as an adjunct to diet and exercise when metformin is unsuitable due to intolerance [6]. This drug has shown significant efficacy in individuals with heart failure (HF) or chronic kidney disease (CKD), offering cardiovascular and renal benefits independent of diabetes status [7]. Due to its specific mechanism of action, dapagliflozin generally does not cause hypoglycemia unless used with medications that cause low blood sugar [8].

Clinical trials have reported a higher incidence of diabetic ketoacidosis (DKA) among patients using SGLT2 inhibitors, including dapagliflozin, than in those using dipeptidyl-peptidase IV (DPP-4) drugs [9,10].

Additionally, SGLT2 inhibitors have been associated with an increased risk of vulvovaginal candidiasis, with incidence rates between 2% and 4% among women with diabetes [11]. Despite the proven benefits of dapagliflozin, ongoing concerns remain regarding its long-term safety, particularly related to diabetic ketoacidosis and genital infections. This study aims to evaluate its safety profile and effectiveness in glycemic control among obese patients with T2DM.

## Materials and methods

Study design and setting

This prospective, multicenter observational study was conducted over 12 months, from April 1, 2023, to April 30, 2024, at BIRDEM (Bangladesh Institute of Research and Rehabilitation in Diabetes, Endocrine and Metabolic Disorders) General Hospital in Dhaka, Bangladesh, and several affiliated accredited healthcare facilities. The study received approval from the Institutional Review Board of BIRDEM (Ref: 11872-BIRDEM/2023) and was conducted in accordance with the Declaration of Helsinki and Good Clinical Practice guidelines. As an observational study, no experimental interventions or protocol-mandated treatments were introduced. All decisions regarding initiation, dose adjustment, and therapeutic combinations were made independently by attending physicians in the context of routine care.

Sampling method and sample size

One thousand five hundred patients were enrolled through a consecutive convenience sampling method from outpatient departments of participating centers. The sample size was considered adequate to facilitate subgroup analyses between dose groups and different therapy combinations, and to evaluate the frequency and patterns of adverse events over time.

Eligibility criteria

Eligible patients were aged between 18 and 75 years, had a diagnosis of type 2 diabetes mellitus based on American Diabetes Association (ADA) 2023 criteria [12], and a body mass index (BMI) of ≥30 kg/m² according to the WHO classification [13]. Inclusion was restricted to those newly initiated on dapagliflozin therapy during the study period. Patients were excluded if they had a history of diabetic ketoacidosis, previous or current use of any other SGLT2 inhibitor, significant renal impairment (eGFR <30 mL/min/1.73 m²), hepatic dysfunction, active urinary or genital tract infection, pregnancy, lactation, or documented contraindications to dapagliflozin.

Treatment protocol and drug regimens

Patients received either 5 mg or 10 mg of dapagliflozin once daily, as prescribed by the treating physician. The choice of dose was made independently by clinicians based on individual patient profiles, clinical judgment, and standard national guidelines from the Directorate General of Drug Administration (DGDA), Bangladesh [14], as well as international recommendations, including the ADA Standards of Care 2023 [12] and European Association for the Study of Diabetes (EASD) [15] consensus statements.

The 10 mg dose was more commonly used and generally preferred in patients requiring greater glycemic or weight control. The 5 mg dose was initiated in selected individuals where physicians considered a lower starting dose more appropriate, such as elderly patients, those at higher risk of adverse events, or those with clinical concerns regarding tolerability. Of the sample, 30 patients received the 5 mg dose, while 1,470 received the 10 mg dose. Dapagliflozin was used either as monotherapy or with non-SGLT2 antidiabetic agents, including metformin, sulfonylureas, DPP-4 inhibitors, and insulin. Based on the number of accompanying agents, patients were classified into monotherapy, dual therapy, triple therapy, or quadruple (or higher-order) therapy groups. No other SGLT2 inhibitors were used in any observed treatment combinations. The treating physicians determined all treatment decisions, including dose adjustments and drug combinations, entirely as part of routine clinical care, without any investigator intervention.

Lifestyle advice

All patients received routine lifestyle counseling during outpatient consultations as part of standard diabetes care. This included guidance on adopting a healthy, low-refined carbohydrate diet, increasing physical activity, and managing body weight. These lifestyle recommendations were not standardized across centers and were not reinforced by the research team; thus, adherence was not assessed as part of the study.

Data sources and collection tools

Clinical and laboratory data were extracted exclusively from medical records, prescription charts, and institutional laboratory reports using a standardized case record form (CRF). Baseline variables included age, sex, BMI, duration of diabetes, comorbidities (e.g., hypertension, dyslipidemia), and concurrent medications. Laboratory parameters included glycated hemoglobin (HbA1c), fasting blood glucose (FBG), and lipid profile components (total cholesterol, LDL, HDL, and triglycerides). While HbA1c and FBG were assessed at baseline, three, six, and 12 months, lipid profiles were recorded explicitly at baseline, six months, and 12 months in line with routine cardiometabolic monitoring practices. All laboratory investigations were conducted at certified institutional laboratories using standardized automated analyzers. A permissible deviation of ±2 weeks was accepted for each scheduled time point.

Adverse event assessment

Adverse events were identified from clinical documentation during routine follow-up visits and included both common and serious complications potentially associated with dapagliflozin use. Commonly recorded events included fatigue, symptomatic hypoglycemia, urinary tract infections, pruritus, and dysuria. Serious adverse events such as diabetic ketoacidosis, Fournier’s gangrene, cellulitis, and testicular abscess were also reviewed for their severity and clinical relevance. All adverse events were classified according to type and suspected association with the drug. Events clearly unrelated to dapagliflozin, such as pneumonia or nephrolithiasis, were noted separately. Therapy discontinuation and the reasons for withdrawal, including adverse effects, were also documented.

Statistical analysis

All statistical analyses were performed using IBM SPSS Statistics for Windows, version 25 (IBM Corp., Armonk, NY). Continuous variables were presented as mean ± standard deviation (SD) or median with interquartile range (IQR), depending on distribution. Categorical data were reported as frequencies and percentages. Between-group comparisons, such as between 5 mg and 10 mg dose groups or across therapy categories, were analyzed using independent sample t-tests or Mann-Whitney U tests. Changes over time in paired variables were assessed using the Wilcoxon signed-rank test. Multivariate logistic regression analysis was employed to identify independent predictors of adverse events, adjusting for patient age, sex, diabetes duration, dapagliflozin dose, and complexity of therapy. Adjusted odds ratios (ORs) with 95% confidence intervals (CIs) were calculated, and a p-value of <0.05 was considered statistically significant.

## Results

A cohort of 1500 individuals was recruited for this study. The majority of participants, 929 (62%), were male. Bangladeshi individuals constituted the majority (1347, 90%), followed by those of Asian origin (119, 8%). The median age of the participants was 52.3 years (IQR: 14.8), and the median BMI was 32.3 kg/m² (IQR: 8.7). The median duration since T2DM diagnosis was 7.83 years (IQR: 5.23). Most participants had multiple comorbidities: 779 (52%) had hyperlipidemia, and 616 (41.1%) had hypertension. Only 30 (2%) participants had experienced a urinary tract infection (UTI) in the year before the study (Table 1).

**Table 1:** Demographic characteristics of obese patients

Parameters	Total number of cases N (%)
Age in years	46.8 ± 8.6
Gender	
Male	930 (62%)
Female	570 (38%)
BMI, kg/m2 (in average)	31.64 ± 6.74
Average duration of type 2 diabetes mellitus (in years)	8.72 ± 5.45
Comorbidity	
Heart failure	60 (4%)
Stroke	30 (2%)
Hypertention	600 (40%)
Hyperlipidemia	780 (52%)
Cardiovascular disorders	60 (4%)

Table 2 compares the baseline characteristics of patients in the 5 mg (n = 30) and 10 mg (n = 1470) dapagliflozin groups. The average age was 53.0 ± 9.5 years in the 5 mg group and 53.6 ± 10.5 years in the 10 mg group (p = 0.73). The mean BMI was 30.4 ± 4.6 kg/m² vs. 31.0 ± 3.8 kg/m² (p = 0.48). Diabetes was 8.0 ± 3.2 years in the 5 mg group and 7.9 ± 2.9 years in the 10 mg group (p = 0.86). Systolic blood pressure was 136.3 ± 17.3 mmHg vs. 135.2 ± 15.0 mmHg (p = 0.73), and diastolic blood pressure was 86.0 ± 10.2 mmHg vs. 85.5 ± 10.3 mmHg (p = 0.79). The baseline HbA1c was slightly higher in the 5 mg group (8.74 ± 0.96%) compared to the 10 mg group (8.50 ± 1.02%), but the difference was not statistically significant (p = 0.18).

**Table 2:** Baseline clinical characteristics by dose group SBP: systolic blood pressure; DBP: diastolic blood pressure; HbA1c: glycated hemoglobin

Characteristic	5 mg group (n = 30)	10 mg group (n = 1470)	t-test value	p-value
Age (years)	53.0 ± 9.5	53.6 ± 10.5	-0.34	0.73
BMI (kg/m²)	30.4 ± 4.6	31.0 ± 3.8	-0.71	0.48
Duration of diabetes (yrs)	8.0 ± 3.2	7.9 ± 2.9	0.17	0.86
SBP (mmHg)	136.3 ± 17.3	135.2 ± 15.0	0.35	0.73
DBP (mmHg)	86.0 ± 10.2	85.5 ± 10.3	0.27	0.79
Baseline HbA1c (%)	8.74 ± 0.96	8.50 ± 1.02	1.35	0.18

A total of 1500 patients received dapagliflozin, with the majority, 1470 (98.1%), prescribed 10 mg for a mean duration of 9.87 ± 2.98 months, while 30 (2%) were prescribed 5 mg for a median duration of 14.23 months. Among them, 1125 (75.1%) patients used supplementary antidiabetic drugs, with metformin being the most commonly prescribed to 953 (84.7%) patients. In terms of combination therapy, dual therapy, which involves dapagliflozin combined with one additional antidiabetic medication, was administered to 600 (40%) patients. In contrast, triple therapy, consisting of dapagliflozin along with two other antidiabetic agents, was used in 450 (30%) patients. Additionally, 90 (6%) patients required more than three antidiabetic medications to achieve glycemic control.

The most significant HbA1c reduction was seen with the triple therapy of dapagliflozin + metformin + sulfonylurea (1.22 ± 0.31%), followed closely by dapagliflozin + metformin only (1.19 ± 0.30%). Weight reduction was similar across most combinations, with the highest in the triple therapy group (3.30 ± 1.20 kg). Fasting blood glucose (FBG) reduction ranged between 32.7 ± 11.5 mg/dL and 35.4 ± 11.2 mg/dL, with the highest reduction seen in the quadruple therapy group (dapagliflozin + metformin + sulfonylurea + DPP-4i/Insulin). Achievement of HbA1c <7% was highest in the quadruple therapy group (73.1%), followed by the Metformin-only combination (70.0%). Adverse events were most frequent with dapagliflozin alone (16.0%) and lowest with the triple therapy (11.3%) and quadruple therapy (11.9%). Overall, combination therapies, especially those including Metformin, were associated with slightly better glycemic control and fewer side effects compared to dapagliflozin alone (Table 3).

**Table 3:** Clinical outcomes by drug combinations DPP-4i: dipeptidyl peptidase-4 inhibitor; FBG: fasting blood glucose; Hba1c: glycated hemoglobin

Drug combination	N (%)	Weight (kg)	HbA1c (%)	FBG (mg/dL)	HbA1c <7%	Adverse events
Dapagliflozin alone	375 (25%)	3.10 ± 1.15	1.15 ± 0.29	34.0 ± 12.0	255 (68.0%)	60 (16.0%)
Dapagliflozin + metformin only	300 (20%)	3.20 ± 1.18	1.19 ± 0.30	34.5 ± 12.3	210 (70.0%)	40 (13.3%)
Dapagliflozin + metformin + sulfonylurea (SU)	400 (26.7%)	3.30 ± 1.20	1.22 ± 0.31	33.8 ± 11.7	275 (68.8%)	45 (11.3%)
Dapagloglozin + metformin + SU + DPP-4i/insulin	253 (16.9%)	3.05 ± 1.25	1.17 ± 0.32	35.4 ± 11.2	185 (73.1%)	30 (11.9%)
Other combos (no metformin)	172 (11.5%)	2.90 ± 1.10	1.10 ± 0.27	32.7 ± 11.5	117 (68.0%)	25 (14.5%)

**Figure 1:**
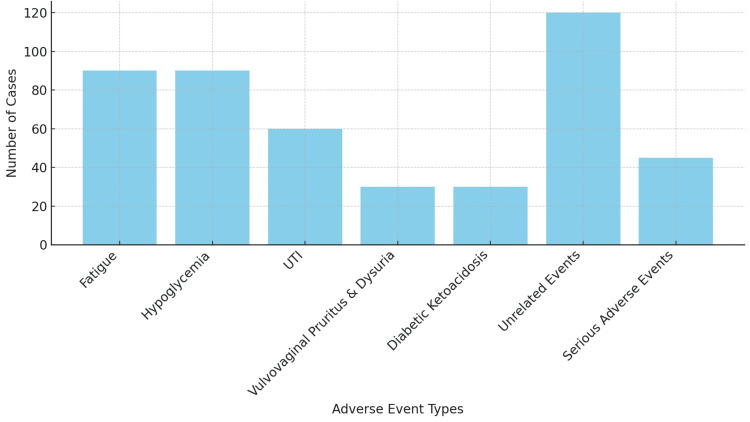
Distribution of adverse events in patients using dapagliflozin UTI: urinary tract infection

Adverse events were reported in 240 (16%) patients, with the most common being 90 (37.5%) cases of fatigue and hypoglycemia each. Urinary tract infection was observed in 60 (25%) cases, vulvovaginal pruritus and dysuria in 30 (12.5%) cases. A total of 30 (12.5%) patients developed diabetic ketoacidosis (DKA), primarily those with a long-standing history of diabetes (≥10 years) and prior hypertension (Figure 1).

Table 4 presents a subgroup analysis of adverse events in the 5 mg (n = 30) and 10 mg (n = 1470) dapagliflozin groups. The overall incidence of adverse events was similar between the two groups, reported in 5 (16.7%) patients in the 5 mg group and 235 (16.0%) in the 10 mg group. Fatigue was observed in two (6.7%) patients in the 5 mg group and 88 (6.0%) in the 10 mg group, while hypoglycemia occurred in the same proportion-2 (6.7%) in the 5 mg group and 88 (6.0%) in the 10 mg group-making both events the most common, each accounting for 90 (37.5%) of total events. Urinary tract infections were reported in one (3.3%) patient in the 5 mg group and 59 (4.0%) patients in the 10 mg group, totaling 60 (25.0%). Pruritus or dysuria was reported only in the 10 mg group in 30 (2.0%) patients, comprising 12.5% of all adverse events. Diabetic ketoacidosis (DKA) was observed in one (3.3%) patient in the 5 mg group and 29 (2.0%) in the 10 mg group, totaling 30 (12.5%). Other unrelated events were recorded in three (10.0%) patients in the 5 mg group and 117 (8.0%) in the 10 mg group, contributing to 120 (50.0%) of all adverse events. Overall, the adverse event profile was broadly comparable between the two dose groups.

**Table 4:** Subgroup analysis of adverse events

Adverse events	5 mg group (N = 30)	10 mg group (N=1470)	Total N (%)
Fatigue	2 (40%)	88 (37.4%)	90 (37.5%)
Hypoglycemia	2 (40%)	88 (37.4%)	90 (37.5%)
Urinary tract infection	1 (20%)	59 (25.1%)	60 (25%)
Pruritus/dysuria	0 (0%)	30 (12.7%)	30 (12.5%)
Diabetic ketoacidosis (DKA)	1 (20%)	29 (12.3%)	30 (12.5%)
Other unrelated events	3 (60%)	117 (49.78%)	120 (50%)
Total events	5 (16.7%)	235 (16%)	240 (16%)

All reported adverse effects were resolved, with 120 (50%) events unrelated to dapagliflozin (e.g., nephrolithiasis, pneumonia, and other comorbid conditions). However, 45 (18.7%) serious adverse events were directly linked to dapagliflozin, including 15 (33.3%) cases of cellulitis, 24 (53.3%) cases of Fournier’s gangrene, and six (13.3%) cases of testicular abscess. Due to adverse effects, 165 (11%) patients discontinued dapagliflozin during the study period.

**Figure 2:**
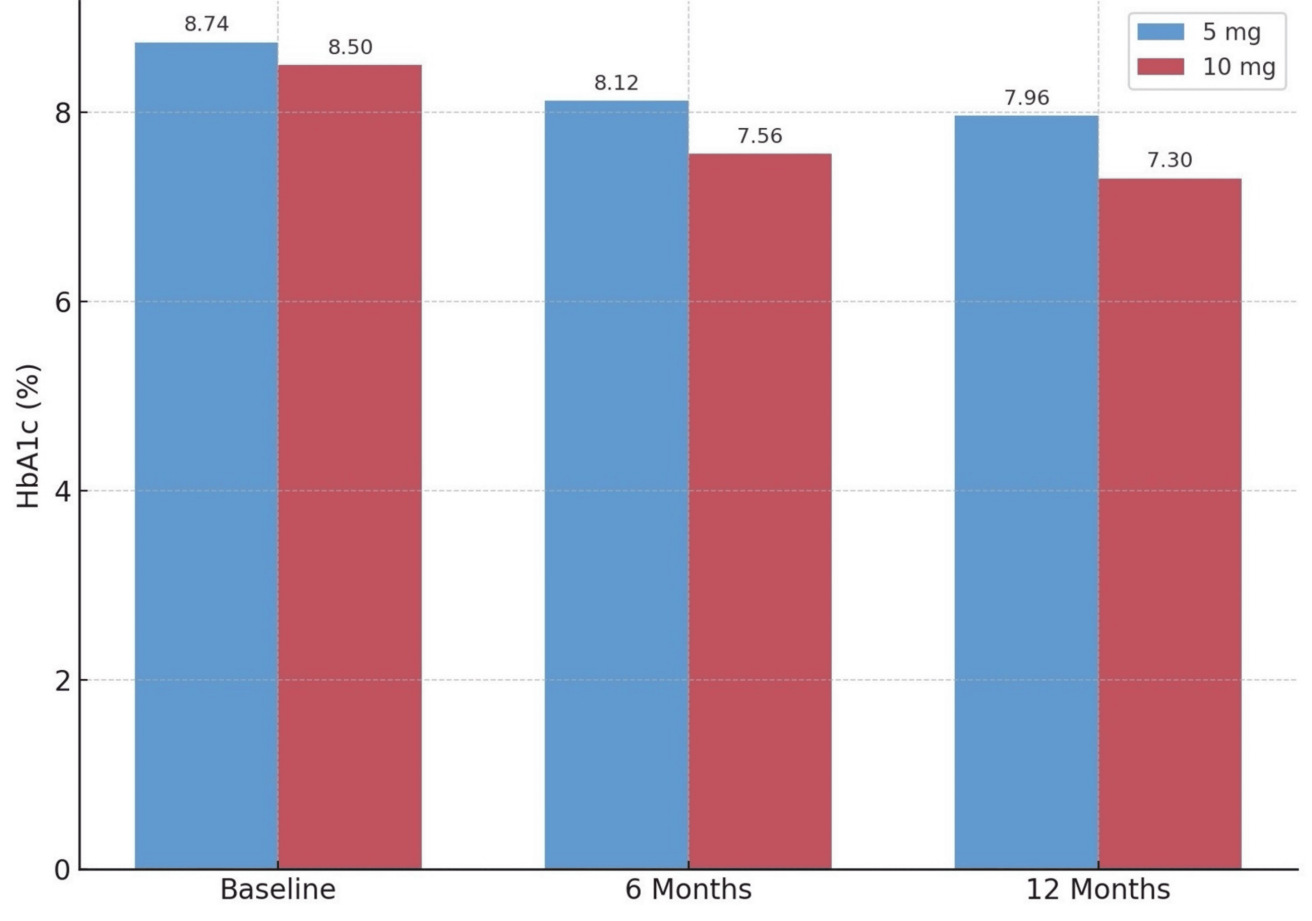
Hemoglobin A1c (HbA1c) levels over time by dose group

The study showed a statistically significant reduction in mean glycated hemoglobin levels from 8.1 ± 1.7 at baseline to 7.3 ± 1.4 after six months of treatment (p<0.0001) either as a standalone therapy or with other anti-diabetic drugs. After a 12-month treatment period, a significant reduction in the average HbA1c% was found compared to the initial HbA1c baseline. The mean HbA1c% decreased to 6.9 ± 1.1 (p < 0.0001). Additional information can be found in Table 5 and Figure 2.

**Table 5:** Hemoglobin A1c (HbA1c) levels of obese patients after dapagliflozin treatment

Variables	Baseline Mean ± SD	After 6 months Mean ± SD	After 12 months Mean ± SD
HbA1c%	8.1 ± 1.7	7.3 ± 1.4	6.9 ± 1.1
Change from baseline	-	-1.07 ± 1.29	-1.20 ± 1.34

Table 6 compares the lipid profile of patients in the 5 mg (n = 30) and 10 mg (n = 1470) dapagliflozin groups. LDL and HDL levels showed statistically significant differences between the two groups. The 10 mg group had lower LDL levels (100 ± 18 mg/dL) compared to the 5 mg group (110 ± 15 mg/dL; p = 0.001), and higher HDL levels (52 ± 11 mg/dL vs. 46 ± 10 mg/dL; p = 0.003), indicating improved lipid outcomes. There was no significant difference in total cholesterol (185 ± 30 mg/dL vs. 176 ± 20 mg/dL; p = 0.113) or triglyceride levels (153 ± 25 mg/dL vs. 150 ± 30 mg/dL; p = 0.52) between the two groups.

**Table 6:** Lipid profile of obese patients by dose group LDL: low-density cholesterol; HDL: high-density cholesterol

Lipid parameter	5 mg group (n = 30)	10 mg group (n = 1470)	t-test value	p-value
Total Cholesterol (mg/dL)	185 ± 30	176 ± 20	1.64	0.113
LDL (mg/dL)	110 ± 15	100 ± 18	3.60	0.001
HDL (mg/dL)	46 ± 10	52 ± 11	-3.25	0.003
Triglycerides (mg/dL)	153 ± 25	150 ± 30	0.65	0.52

Table 7 compares lipid profile parameters across various dapagliflozin-based drug combinations. Total cholesterol levels were relatively consistent across the groups, ranging from 174 ± 21 mg/dL in the quadruple therapy group (dapagliflozin + metformin + sulfonylurea + DPP-4i/insulin) to 178 ± 23 mg/dL in combinations without metformin. Low-density lipoprotein (LDL) levels were lowest in the quadruple therapy group (97 ± 20 mg/dL) and highest among patients receiving other combinations without metformin (103 ± 19 mg/dL). High-density lipoprotein (HDL) levels showed a positive trend with more intensive combination therapies, with the highest value observed in the quadruple therapy group (54 ± 13 mg/dL), followed by the triple therapy group (53 ± 12 mg/dL). The lowest HDL level was recorded in the group without Metformin (50 ± 11 mg/dL). Triglyceride levels remained broadly comparable across all groups, ranging from 147 ± 27 mg/dL to 151 ± 31 mg/dL, with minimal variation. Overall, patients receiving combination therapies that included metformin (particularly triple and quadruple regimens) demonstrated slightly more favorable lipid profiles, especially in terms of LDL reduction and HDL elevation, compared to those receiving dapagliflozin alone or in non-metformin regimens.

**Table 7:** Lipid profile of obese patients by drug combinations DPP-4i: dipeptidyl peptidase-4 inhibitor; LDL: low-density cholesterol; HDL: high-density cholesterol

Drug combination	Total cholesterol (mg/dL)	LDL (mg/dL)	HDL (mg/dL)	Triglycerides (mg/dL)
Dapagliflozin alone	175 ± 22	102 ± 17	51 ± 10	148 ± 28
Dapagliflozin + metformin only	176 ± 20	100 ± 18	52 ± 11	150 ± 30
Dapagliflozin + metformin + sulfonylurea (SU)	177 ± 19	99 ± 17	53 ± 12	149 ± 29
Dapagliflozin + metformin + SU + DPP-4i/insulin	174 ± 21	97 ± 20	54 ± 13	151 ± 31
Other combos (no metformin)	178 ± 23	103 ± 19	50 ± 11	147 ± 27

After adjusting for potential confounding variables, including therapy type, duration of diabetes, age, and gender, the multivariate logistic regression analysis identified several significant predictors of adverse events. The use of dapagliflozin 10 mg was not independently associated with an increased risk of adverse events compared to the 5 mg dose (OR: 1.34; 95% CI: 0.78-2.29; p = 0.28), suggesting that higher dosage alone does not contribute to adverse outcomes. However, the type of therapy demonstrated a dose-dependent association with adverse event risk. Patients receiving dual therapy had a 42% higher likelihood of experiencing adverse events compared to those on monotherapy (OR: 1.42; 95% CI: 1.01-1.99; p = 0.04). This risk increased further in patients on triple therapy (OR: 1.75; 95% CI: 1.30-2.37; p < 0.001) and was highest among those receiving more than triple therapy (OR: 2.50; 95% CI: 1.72-3.63; p < 0.001), indicating a strong association between polypharmacy and the occurrence of adverse events. Duration of diabetes also emerged as a significant predictor, with each additional year of disease increasing the odds of adverse events by 8% (OR: 1.08; 95% CI: 1.04-1.12; p < 0.001). In contrast, neither age (OR: 1.01; 95% CI: 0.99-1.02; p = 0.34) nor gender (OR: 1.10; 95% CI: 0.84-1.45; p = 0.49) showed a significant independent association with adverse event risk in the adjusted model (Table 8).

**Table 8:** Predictors of adverse events (multivariate analysis) * shows significant p-value; p-value less than 0.05 was considered significant

Predictor	Odds ratio (OR)	95% confidence interval	p-value
10 mg dose vs 5 mg dose	1.34	0.78 – 2.29	0.28
Dual therapy vs mono therapy	1.42	1.01 – 1.99	0.04*
Triple therapy	1.75	1.30 – 2.37	<0.001*
> Triple therapy	2.50	1.72 – 3.63	<0.001*
Duration of diabetes	1.08	1.04 – 1.12	<0.001*
Age (per year increase)	1.01	0.99 – 1.02	0.34
Male vs female	1.10	0.84 – 1.45	0.49

## Discussion

This study provided real-world insights into the effectiveness and safety of dapagliflozin in obese Bangladeshi patients with type 2 diabetes (T2DM). The findings highlighted key trends in glycemic control, side effects, and metabolic changes, offering valuable perspectives for clinicians managing similar populations in South Asia.

The study cohort was predominantly male (62%), with a median age of 52.3 years and a high BMI (32.3 kg/m²), reflecting the growing epidemic of obesity and early-onset diabetes in South Asia [16,17]. Many patients had long-standing diabetes (median duration: 7.83 years) and coexisting conditions like hypertension (41.1%) and hyperlipidemia (52%). This aligns with regional studies showing that South Asians often develop diabetes at a younger age and with more severe metabolic complications compared to Western populations [18]. Interestingly, only 2% of participants reported prior urinary tract infections (UTIs), which is lower than rates seen in previous studies [19,20]. This could be due to differences in reporting habits, genetic factors, or lifestyle influences unique to this population.

Dapagliflozin demonstrated strong efficacy in lowering HbA1c, with levels decreasing from 8.1% at baseline to 6.9% after 12 months (p < 0.0001) [21]. The most significant reductions were seen in patients on triple therapy (dapagliflozin + metformin + sulfonylurea), where HbA1c fell by 1.22%. This supports previous South Asian research, where combination therapies with SGLT2 inhibitors and metformin showed increased glycemic benefits compared to monotherapy [22]. Notably, the 10 mg dose of dapagliflozin was not significantly more effective than the 5 mg dose [23], suggesting that higher doses might not always be necessary for optimal blood sugar control in this group.

A critical yet under-discussed finding was the consistent weight loss across all treatment groups (2.9-3.3 kg), with triple therapy showing the most significant reduction (3.3 kg). This is clinically significant for South Asian populations, where even modest weight loss (5-7%) improves insulin sensitivity and cardiovascular risk, a key concern given this cohort's median BMI of 32.3 kg/m² [24]. Notably, the weight loss occurred despite high baseline insulin use (26.9% of participants), contrasting with earlier beliefs that SGLT2 inhibitors might be less effective in insulin-treated patients [25]. The similar weight reductions between 5 mg and 10 mg doses (3.1 vs. 3.0 kg, p=0.48) suggested that lower doses might suffice for metabolic benefits in this population, potentially reducing cost burdens, a relevant factor for resource-limited settings [20]. These findings align with recent South Asian consensus guidelines advocating SGLT2 inhibitors as dual-purpose therapy for both glycemic control and weight management in obese diabetics [26].

About 16% of patients experienced side effects, with fatigue (37.5%) and hypoglycemia (37.5%) being the most common. UTIs occurred in 25% of cases, higher than in some global trials [20] but lower than in other South Asian reports [27]. This variation could stem from healthcare access, antibiotic use, or genetic susceptibility differences.

A concerning finding was the relatively high rate of diabetic ketoacidosis (DKA) (12.5%), which exceeds rates seen in previous studies (typically 1-2%) [28]. This might be linked to the higher prevalence of insulin deficiency and prolonged diabetes duration in South Asian patients. Severe complications like Fournier’s gangrene, though rare, were also observed, reinforcing the need for careful monitoring in high-risk individuals [29].

Patients on the 10 mg dose showed better lipid outcomes than those on 5 mg, with significantly lower LDL (100 vs. 110 mg/dL, p-value=0.001) and higher HDL (52 vs. 46 mg/dL, p-value=0.003). The best results were seen in patients on quadruple therapy (dapagliflozin + metformin + sulfonylurea + DPP-4i/insulin), suggesting that intensive combination regimens might offer additional cardiovascular benefits. These findings are consistent with recent studies, where SGLT2 inhibitors improved lipid profiles in obese diabetics [30,31].

Multivariate analysis revealed that patients on multiple diabetes medications (especially triple or quadruple therapy) had a higher risk of adverse events. Each additional year of diabetes also increased the likelihood of complications by 8%. However, neither age nor gender significantly influenced risk, which contrasts with some global data [32] but aligns with South Asian studies where metabolic factors play a bigger role than demographics [33].

Nevertheless, dapagliflozin is an effective option for obese South Asian patients with T2DM, particularly when combined with metformin. However, the higher rates of DKA and UTIs highlighted the need for careful patient selection and monitoring. These findings supported personalized treatment approaches in this high-risk population, balancing glycemic benefits with potential risks. As an observational study, these results cannot prove causation, and the lack of a control group limits direct comparisons. However, the real-world setting provides practical insights for clinicians treating similar patients in South Asia, where clinical trial data is often scarce.

Limitations

This study has several limitations. First, due to its prospective observational design, it lacks a control group, which limits causal inference and makes it difficult to attribute observed outcomes solely to dapagliflozin. Second, although adverse events were documented during clinical follow-ups, no formal causality assessment tools were used. Also, tolerability was not evaluated using standardized patient-reported questionnaires, potentially leading to underestimation or misclassification of some events. Third, essential confounders such as the concomitant use of statins or detailed dietary adherence (e.g., fiber and protein intake) were not consistently recorded, which may have influenced lipid outcomes and other metabolic parameters. Fourth, the diagnosis of urinary tract infections was based on physician assessment, and urine routine or culture tests were not uniformly performed, potentially affecting diagnostic accuracy. Fifth, although some cases of diabetic ketoacidosis (DKA) were observed and were likely multifactorial, glucose levels and ketone status were not uniformly available to identify euglycemic DKA in all cases. Lastly, the sample was drawn from hospital outpatient clinics using convenience sampling, which may introduce selection bias and limit the generalizability of the findings to the broader diabetic population.

## Conclusions

This study provides valuable real-world evidence supporting the clinical efficacy and safety of dapagliflozin in the management of obese patients with type 2 diabetes mellitus. The findings demonstrate that dapagliflozin contributes significantly to improvements in glycemic control, as evidenced by sustained reductions in HbA1c levels over the 12-month follow-up period. Additionally, favorable trends were observed in weight reduction and lipid profile modulation, particularly among patients receiving combination therapies that included metformin and sulfonylureas. Although adverse events were reported, including hypoglycemia, fatigue, urinary tract infections, and diabetic ketoacidosis, the majority were non-severe and manageable through standard clinical interventions. Importantly, multivariate analysis revealed that the incidence of adverse events was more strongly associated with longer diabetes duration and use of multiple antidiabetic agents, rather than the dosage of dapagliflozin itself. This suggests that appropriate patient selection and consideration of treatment complexity may help mitigate risk. The results further reinforce the clinical value of dapagliflozin as a monotherapy and as part of a broader pharmacologic regimen tailored to individual patient needs.
